# Integrating Unknown Metabolites to Optimize a Metabolomic Distress Score for Psychological Distress in Older Women

**DOI:** 10.1093/geroni/igaf122.4412

**Published:** 2025-12-31

**Authors:** Yiwen Zhu, Raji Balasubramanian, Julián Ávila-Pacheco, Clary Clish, Jie Yao, Jerome Rotter, Laura Kubzansky, Susan Hankinson

**Affiliations:** Harvard T.H. Chan School of Public Health, Boston, Massachusetts, United States; University of Massachusetts Amherst, Amherst, Massachusetts, United States; Broad Institute of MIT and Harvard, Cambridge, Massachusetts, United States; Broad Institute of MIT and Harvard, Cambridge, Massachusetts, United States; The Lundquist Institute at Harbor-UCLA Medical Center, Torrance, California, United States; The Lundquist Institute at Harbor-UCLA Medical Center, Torrance, California, United States; Harvard T.H. Chan School of Public Health, Boston, Massachusetts, United States; University of Massachusetts Amherst, Amherst, Massachusetts, United States

## Abstract

Chronic distress, including depression and anxiety, contributes significantly to cardiometabolic risk in aging populations, with metabolic alterations acting as a key pathway. We previously developed a metabolomic distress score (MDS) using 20 identified metabolites in older White women, which predicted cardiometabolic risk in aging cohorts. Here, we expand this approach by identifying metabolomic features not yet characterized that are associated with distress and derive a revised score incorporating these features (rMDS). Assays were conducted at the Broad Institute using nontargeted liquid chromatography-mass spectrometry methods. We performed agnostic analyses assessing associations between distress and up to 5539 unannotated features among older White women from three cohorts: Nurses’ Health Study (N = 540, mean age=64), Women’s Health Initiative Observational Cohort (N = 1107; mean age=67), and Multi-Ethnic Study of Atherosclerosis (N = 770; mean age=63). We selected unknown metabolites and derived an rMDS via the least absolute shrinkage and selection operator (LASSO). We identified 14 features robustly associated with distress across all cohorts (p < 0.1), plus 65 features in the two most recently assayed cohorts. Ongoing biochemical identification has led to 26 new metabolite annotations among the features identified, including cholesteryl esters and zeaxanthin. The revised distress score (rMDS) integrated novel signals from 67 LASSO-selected known and unknown metabolites. In NHS, rMDS was more strongly associated with distress than the MDS (OR = 5.15, 95%CI [3.85-7.06] vs. OR = 3.30, 95%CI [2.59-4.27]). Validation of rMDS in independent samples is ongoing. Incorporating previously unidentified metabolomic features greatly enhanced the performance of the metabolomic score, revealing novel pathways linking psychological distress to health and aging.

